# Secular trends in age at pubertal onset assessed by breast development among Chinese girls: A systematic review

**DOI:** 10.3389/fendo.2022.1042122

**Published:** 2022-11-24

**Authors:** Wen Shu, Xin’nan Zong, Hui Li

**Affiliations:** ^1^ Department of Growth and Development, Capital Institute of Pediatrics, Beijing, China; ^2^ Peking Union Medical College, Chinese Academy of Medical Sciences, Beijing, China

**Keywords:** girls, adolescents, breast development, precocious puberty, China

## Abstract

**Background:**

The average age at thelarche has trended downwards worldwide since 1970s; however, the onset age of “precocious puberty”, defined as the lower percentiles of thelarche age, has been rarely reported. This systematic review aims to evaluate secular trends in age at thelarche among Chinese girls.

**Methods:**

This systematic review on the age at thelarche during puberty among Chinese girls was conducted *via* systematic search of both Chinese (Chinese National Knowledge Infrastructure, WanFang Database, and the Chinese Scientific Journals Database) and English (PubMed, Cochrane Library, and Embase) databases. Data were analyzed using the GraphPad Prism v9.0.

**Results:**

A total of 16 studies involving 177,886 Chinese girls were synthesized. The QualSyst scores of these studies were high at an average of 21.25. The timing of Tanner breast stage 2 (B2) occurred earlier over time at the P_3_, P_10_, and median ages. Weighted analyses revealed that the overall onset age of B2 tended to be younger at P_3_, P_10_, and P_25_. The age of B2 varied across regions and areas. For example, P_3_, P_10_, and median age of B2 in years were younger in southern regions than that in northern regions of China (P_3_: 5.94 *vs*. 7.3; P_10_: 6.6 *vs*. 7.9; median age: 8.26 *vs*. 9.5), and median age of B2 in urban areas (8.26 years) was earlier than that in rural areas (10.29 years). In addition, median age of B2 from 12 single-center studies was earlier than that from 4 multicenter studies (8.26 *vs*. 9.18 years).

**Conclusions:**

The current findings indicated that pubertal breast development age among Chinese girls presented an advanced trend over the past 20 years, which urges the necessity to revisit and redefine “precocious puberty” and provides useful recommendations for clinical practice.

## 1 Introduction

Adolescence is a major developmental stage of life, and it includes profound physical, psychosocial, and cognitive changes (1). Thelarche is a critical clinical milestone in the onset of puberty. According to the Tanner scale stages, visual inspection and breast palpation are regarded as the current gold standards for clinical evaluation of a girl’s progress through puberty (1, 2).

The onset of puberty is initiated by the reactivation of the previously suppressed hypothalamic-pituitary-gonadal (HPG) axis, and it is influenced by genetic, nutritional, and environmental factors (3). Over the last 20 years, several large-scale studies from high-income countries have reported earlier onset of puberty in girls than historical norms due to improved nutrition and changing environment (4). A similar trend has also been observed in low- and middle-income countries (1). Recently, Eckert-Lind and colleagues have written an excellent systematic review and meta-analysis, and demonstrated that age at thelarche decreased by almost 3 months per decade from 1977 to 2013 around the world (5). Additionally, many studies also supported early occurrence at the onset of puberty (6).

Globally, trend studies on pubertal onset are rarely reported in lower percentile ages (for example, 3^rd^ [P_3_] and 10^th^ percentile [P_10_]), which restricts precise assessment of whether pubertal development was advanced in girls. Although some studies in sporadic or local areas of China have demonstrated that girl breasts tended to develop earlier (7, 8), national data on pubertal onset age are yet lacking.

This systematic review evaluated the overall secular changes in age at pubertal onset measured by age at thelarche among Chinese girls. The current findings would provide evidence for scientific research on pubertal development worldwide and a reference for revisiting or redefining “precocious puberty” in Chinese girls.

## 2 Methods

### 2.1 Data sources and search strategy

We conducted comprehensive literature search on the Chinese National Knowledge Infrastructure (CNKI), WanFang Database, Chinese Scientific Journals Database (VIP), PubMed, Cochrane Library, and Excerpt Medica Database (EMBASE) from their inception to April 2022. The following key terms expressed in the Boolean form were used to search related articles: [((puberty) OR (pubertal) OR (adolescent) OR (adolescence)) AND (sexual)]]. No language restrictions were imposed. The detailed search strategy is shown in **Supplementary Table S1**.

### 2.2 Inclusion and exclusion criteria

Studies that satisfied the following criteria simultaneously were included in this review: 1) healthy girls from population surveys; 2) no exposure factors and no intervention factors; 3) available onset age and percentiles of Tanner breast stage 2 (B2); 4) usage of the Tanner scale stages to evaluate development process of secondary sexual characteristics.

Studies were excluded if any one of the following criteria was met: 1) case-control studies, case reports, or review articles; 2) articles without age of Tanner breast stage 2 (B2) onset and percentile; 3) repeated reports.

### 2.3 Data extraction

Two investigators (WS and XNZ) extracted data independently using a standardized data extraction form. All records identified were managed by the EndNote software version X9, and duplicate articles were removed. Titles and abstracts of identified articles were screened independently. If necessary, full texts of articles were read thoroughly to assess their eligibility. A third investigator (HL) was consulted in case of disagreement, and a 100% consensus was reached.

Data were entered into the Microsoft Excel 365, including the first author, time of study, study location, range of age, sample size, median age and percentile of B2, as well as breast development staging methods (questionnaire/visual and palpation/physical examination), if available.

### 2.4 Quality assessment

The QualSyst quality assessment tool was used to assess risk of bias, and each study was assessed by 14 criteria (9). Each criterion was scored at both study and outcome levels, depending on the degree to which specific criteria were met or reported (“yes” = 2; “partial” = 1; “no” = 0). The items not applicable to a particular study design were marked “N/A” and excluded from the calculation of the summary score. A total score of 22 points was set for quality evaluation according to the design of this systematic review. The studies were categorized as strong (>75%), moderate (55-75%), and weak quality (<55%). The quality of included articles was independently assessed by two investigators (WS and XNZ). Any disagreement was resolved until a 100% consensus was reached.

### 2.5 Data synthesis

The bubble plot of P_3_, P_10_, P_25_, and median age of B2 in different periods was drawn using the GraphPad Prism software version 9.0. Results of the literature from the same period were weighted for analysis and plotted using this software.

## 3 Results

### 3.1 Search summaries

**Figure 1:**
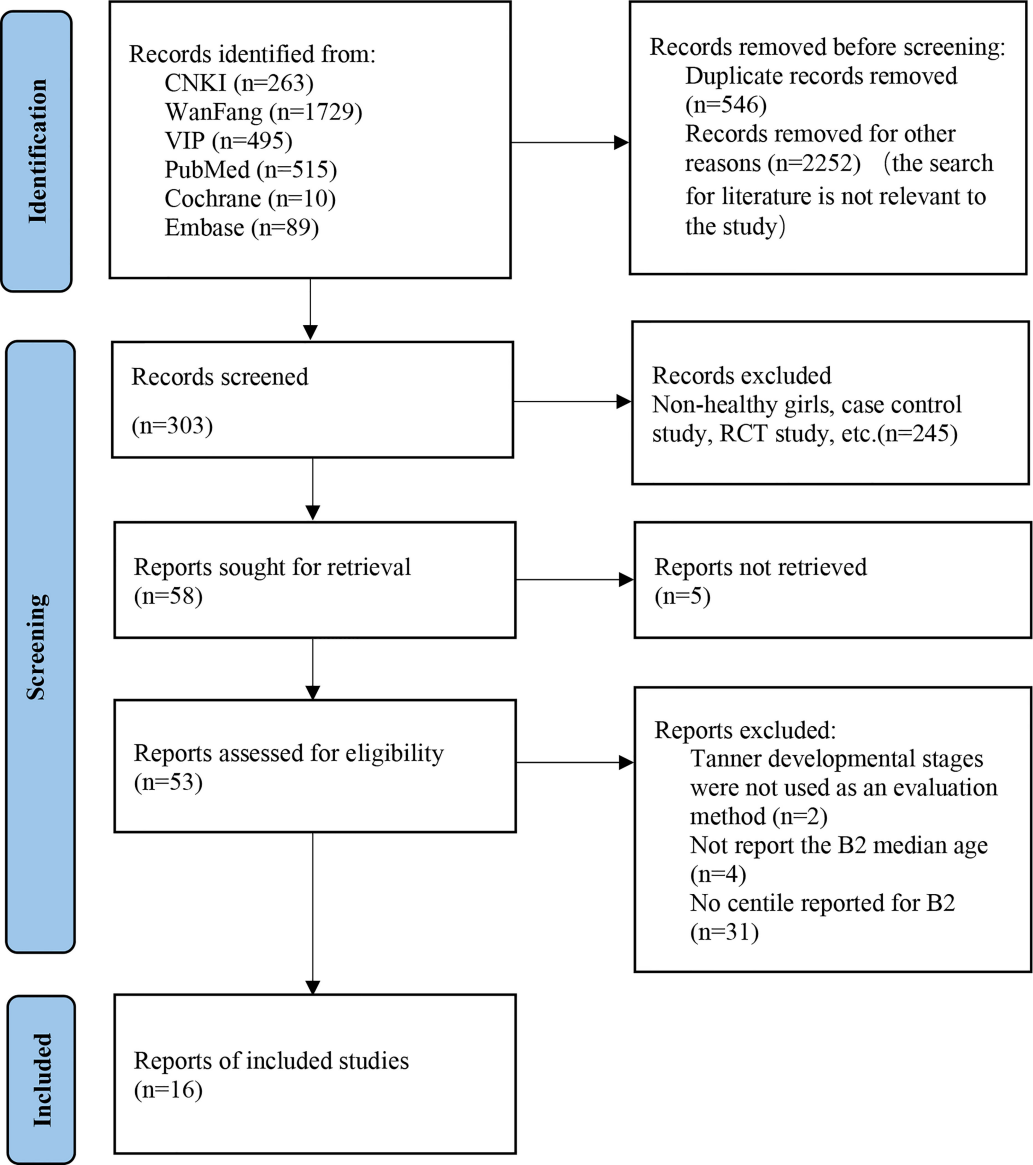
The PRISMA flow diagram.

The initial search yielded 3,101 potential articles. After removing duplicates and unrelated articles, 303 were reviewed. Subsequently, 58 articles were included after scanning the titles and abstracts. The full-texts of the remaining 53 articles were read, and of them 37 articles were excluded due to the lack of Tanner stages to determine the development level of secondary sexual characteristics or age of B2 onset and percentiles information. Finally, the results of the remaining 16 independent articles were synthesized in this systematic review (10–24). The PRISMA flowchart of articles that were excluded with specific reasons is shown in **Figure 1**.

### 3.2 Assessment of bias

The study quality was assessed for risk of bias using the QualSyst tool (**Table 1**). The average score was 21.25 (range: 20 to 22) points. The common limitations in the included studies were insufficient controlling for confounding factors, insufficient subject selection strategy (sampling frame) or source of information described, and analytic methods not described in detail.

**Table 1:** Literature quality asssement tool: QualSyst tool.

Quality assessment criteria	Pu Jiaqi, 2021	Guan Peiyu, 2016	Luo Xuan, 2018	Yang Mingzhe, 2015	Sun Ying, 2013	Fang Qi, 2012	Li Dan, 2013	Gu Kefeng, 2013	Wang Zhen, 2011	Yan Mei, 2012	Zhu Minqiang, 2013	Yang Yajuan, 2010	Hou Dongqing, 2006	Luo Xuan, 2018	The Pubertal Study Group of the Society of Pediatric Endocrinology and Genetic Disease, Chinese Medical Association, 2010	KF Huen, 1997
(1)	2	2	2	2	2	2	2	2	2	2	2	2	2	2	2	2
(2)	2	2	2	2	2	2	2	2	2	2	2	2	2	2	2	2
(3)	2	2	2	2	2	2	2	1	2	2	2	2	2	2	1	2
(4)	2	2	2	2	2	2	2	2	2	2	2	2	2	2	2	2
(5)	N/A	N/A	N/A	N/A	N/A	N/A	N/A	N/A	N/A	N/A	N/A	N/A	N/A	N/A	N/A	N/A
(6)	N/A	N/A	N/A	N/A	N/A	N/A	N/A	N/A	N/A	N/A	N/A	N/A	N/A	N/A	N/A	N/A
(7)	N/A	N/A	N/A	N/A	N/A	N/A	N/A	N/A	N/A	N/A	N/A	N/A	N/A	N/A	N/A	N/A
(8)	2	2	2	2	2	2	2	2	2	2	2	2	2	2	2	2
(9)	2	2	2	2	2	2	2	2	2	2	2	2	2	2	2	2
(10)	2	2	2	2	1	2	2	2	2	2	2	2	2	2	2	2
(11)	2	2	2	2	2	2	2	2	2	2	2	2	2	2	2	2
(12)	2	2	2	1	2	1	2	1	1	2	1	1	1	2	1	1
(13)	2	2	2	2	2	2	2	2	2	2	2	2	2	2	2	2
(14)	2	2	2	2	2	2	2	2	2	2	2	2	2	2	2	2
TOT(/22)	22	22	22	21	21	21	22	20	21	22	21	21	21	22	20	21

green, 2 points; yellow, 1 point; N/A, Not applicable.

### 3.3 Breast onset time in puberty

#### 3.3.1 Classification according to time

**Figure 2:**
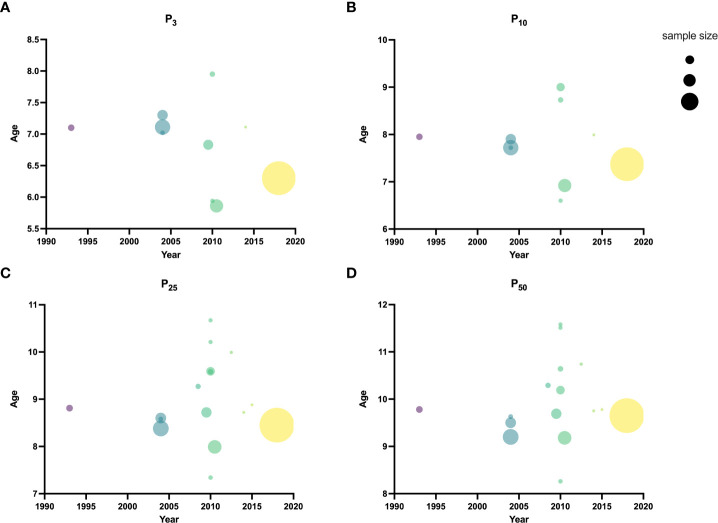
Ages for each time period of P_3_ **(A)**, P_10_ **(B)**, P_25_ **(C)**, and P_50_ **(D)** of B2.

**Figure 3:**
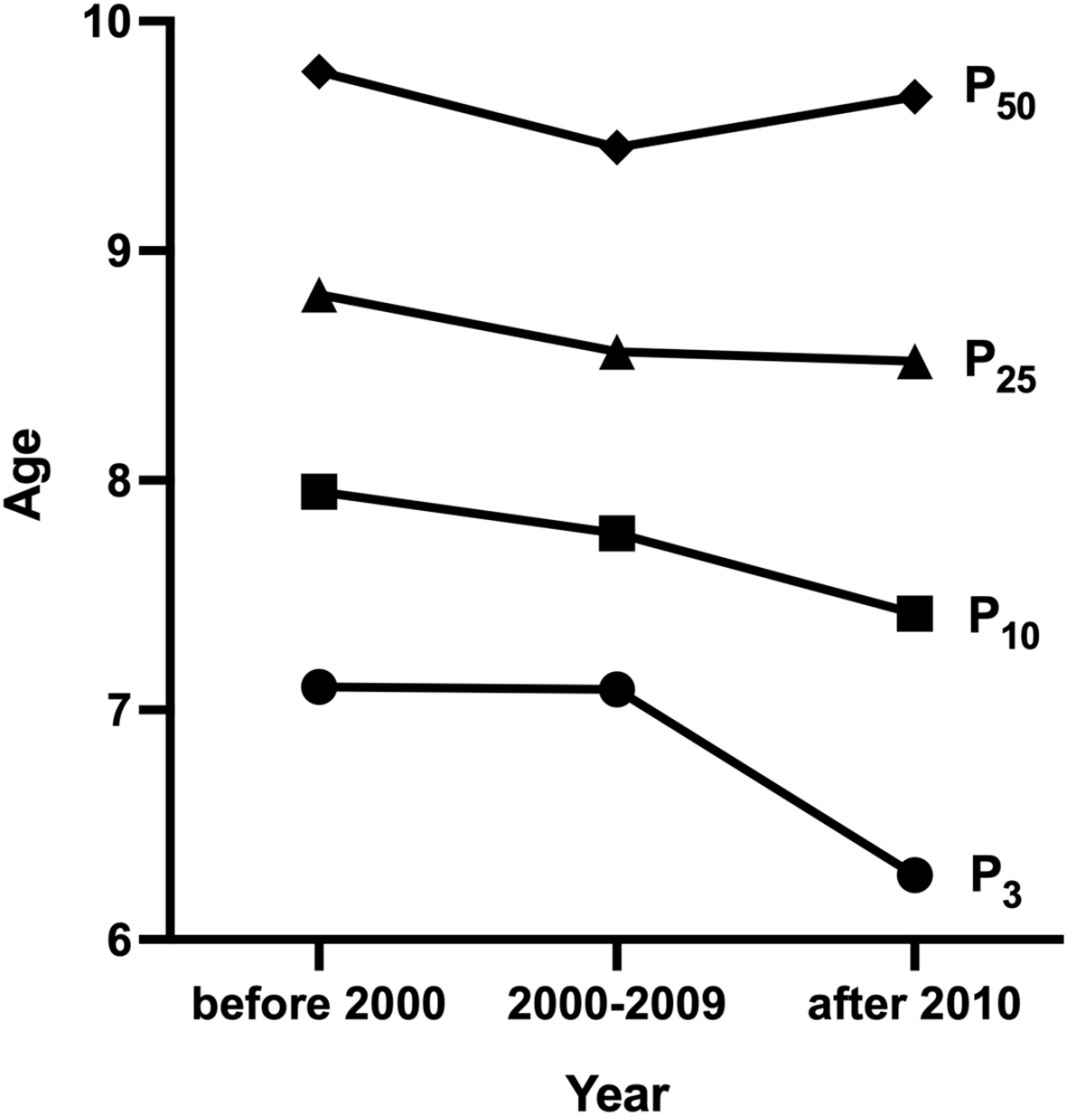
Weighted analysis of P_3_, P_10_, P_25_ and median age for B2.

One research was conducted before 2000 (23), 5 researches from 2000 to 2009 (18–22), and 10 after 2010 (10–17, 21). The median age at B2 was 9.78 [95% confidence interval (CI): 9.70-9.85] years before 2000. The lowest B2 median ages were 9.20 (95% CI: 9.06-9.32) and 8.26 (95% CI: 8.10-8.42) years from 2000-2009 and after 2010, respectively. Before 2000, the P_3_ and P_10_ ages of B2 were 7.1 and 7.95 years, respectively. The lowest P_3_ age of B2 was 6.83 years, and the lowest P_10_ was 7.72 years from 2000-2009. The lowest P_3_ and P_10_ of B2 after 2010 were 5.86 (95% CI: 5.17-6.39) and 6.6 years, respectively (**Table 2** and **Figure 2**). Weighted analyses over the same period revealed that P_3_, P_10_, and P_25_ for B2 decreased significantly, while median age exhibited a slight increase after 2010 (**Figure 3**).

**Table 2:** Characteristics of the included studies.

Studies	Study location	Study period (year)	Age range	Sample size	P_3_	P_5_	P_10_	P_25_	P_50_	P_75_	P_90_	P_95_	P_97_	Examination/Questionnaire
Pu JQ	Jilin, Beijing, Tianjin, Shanghai, Zhejiang, Hubei, Henan, Guangdong, Fujian, Guangxi, Chongqing, Xinjiang	2017-2019	3-18	99694	6.3	6.72	7.37	8.45	9.65 (9.63-9.68)	10.86	11.94	12.59	13.01	Examination^a^
Guan PY	Chongqing	2015	7-17	672				8.88	9.78	10.77				Examination
Luo X	Chongqing	2013-2015	6-18	714	7.11		7.99	8.72	9.75	10.97	11.98		12.88	Palpation
Yang MZ	Chengdu	2012-2013	7-16	890				9.99	10.74	11.59				Examination^a^
Sun Y*	Shenyang, Shanghai, Chongqing, Guangzhou, Hefei, Zhengzhou, Tianjin, Wuhan	2010-2011	6-18.9	15388	5.86 (5.17-6.39)		6.92 (6.39-7.33)	7.99 (7.60-8.31)	9.18 (8.91-9.44)	10.32 (10.10-10.67)	11.44 (11.10-11.87)		12.50 (12.06-13.07)	Examination
Fang Q	Chongqing	2010	8-19	1470				10.21	11.51	13.11				Examination
Li D*	Shanghai	2010	6-18	1675	5.94		6.6	7.34	8.26 (8.10-8.42)	9.3	10.35		11.5	visual inspection
Gu KF	Suzhou	2010	6-12	6135		8.58	9	9.59	10.17	10.92	11.42	11.68		Examination^a^
Wang Z	Zhengzhou	2010	7-18	2585	7.95		8.73	9.58	10.64	11.8	12.96		14.22	Examination^a^
Yan M	Wuhan	2010	9-17	1429				10.67	11.58	12.53				Examination
Zhu MQ*	Beijing, Tianjin, Hangzhou, Shanghai, Chongqing, Nanning	2009-2010	6-18	8895	6.83			8.72	9.69 (9.63-9.75)	10.67			12.42	Examination^a^
Yang YJ	Anhui	2008-2009	8-17	2372				9.27	10.29	11.42				Questionnaire
Hou DQ	Beijing	2004	6-18	9678	7.3	7.5	7.9	8.6	9.5	10.4	11.3	11.9	12.3	Examination^a^
The Pubertal Study Group of the Society of Pediatric Endocrinology and Genetic Disease, Chinese Medical Association	Beijing, Tianjin, Qingdao, Shanghai, Wuhan, Nanning, Chongqing, Guangzhou, Fuzhou	2003-2005	3-19.8	20654	7.11		7.72	8.38	9.20 (9.06-9.32)	10.08	10.95		11.89	Examination^a^
Luo X	Chongqing	2003-2005	6-17	1886	7.02		7.72	8.58	9.63	10.67	11.55		12.32	Palpation
KF Huen*	Hong Kong	1993	7-19	3749	7.1		7.95	8.81	9.78 (9.70-9.85)	10.74	11.6		12.26	Examination

^*^the literature gives a 95% confidence interval for B2 percentile age. ^a^Palpation and visual inspection.

#### 3.3.2 Classification according to locations

Most of included studies were performed in southern regions of China. In northern regions, the lowest median age of 9.5 years at B2 was reported in Beijing and in southern regions, the lowest median age of 8.26 years at B2 was reported in Shanghai. In northern regions, the lowest P_3_ and P_10_ for B2 were 7.3 and 7.9 years, respectively based on a study in Beijing, while that in southern regions were 5.94 and 6.6 years respectively from a study in Shanghai.

#### 3.3.3 Classification according to populations

A larger number of girls were investigated in urban than in rural areas, and two studies reported the median age of B2 in both urban and rural areas (12, 25). The lowest B2 median age was 8.26 years in girls from urban areas, and 10.29 years from rural areas. This review consisted of more single-center studies than multicenter studies. The largest number of single-center and multicenter populations were from Beijing and nine cities, and the lowest median age of B2 from single-center and multicenter studies was 8.26 and 9.18 years, respectively.

All the studies included overweight and obese girls, and only one article conducted the probabilistic regression of breast stage for overweight and obese girls (12).

## 4 Discussion

To the best of our knowledge, this is the first systematic assessment of secular trends on the timing of pubertal onset among Chinese girls based on age of breast development. The key finding of this systematic review is that the pubertal breast development age of Chinese girls seems to present an advanced trend over the past 20 years.

It is worth noting that we here observed a trend of early breast development in Chinese girls at both median age and P_3_ and P_10_ of B2. The median age of B2 advanced by 1.52 years from 1990s to 2010s (9.78 years in 1993 versus 8.26 years in 2010). P_3_ and P_10_ for B2 ranged from 7.1-7.95 years in 1993 to 5.86-6.6 years in 2010, which advanced by 1.24 and 1.35 years, respectively. Weighted analyses over the same period showed that P_3_ and P_10_ of B2 decreased significantly over time. Studies from many other countries have found that age at thelarche onset was advanced. The Pediatric Research in the US Office Setting (PROS) indicated that the timing of breast development in white American girls was advanced from 10.6 years in 1930s-1940s to 9.96 years in 1992-1993 (26). Another finding revealed that the age of B2 among girls in Denmark was 9.86 years in 2006-2008, nearly a year earlier than the 10.88 years reported in 1992-1993 (27). Atay and colleagues analyzed the median age of breast development in 4,868 healthy girls aged 6-18 years in Turkey in 2009 and compared to data from 40 years ago, and they also observed a downward trend in the median age of breast development (28). Improvement in the socioeconomic and nutritional status may be the primary factor behind early puberty development (12). The present review showed that more studies on breast development of girls in the south than in the north of China, and the timing of breast development differed between the two regions. The lowest B2 median, P_10_, and P_3_ ages were 8.26, 6.6, and 5.94 years in southern regions, earlier than 9.5, 7.9, and 7.3 years in northern regions. This phenomenon may be attributable to the differences in economic development, living environment, atmospheric environment, and dietary habits between the north and south of China (18). Also, we observed that breast development of urban girls (8.26 years) began earlier than that of rural girls (10.29 years). This observation might be related to the differences in nutritional status, food composition, or dietary intake of nutrients among urban and rural girls (29, 30). For example, total fat and protein intake, which are relatively higher in urban girls than in rural girls, contribute to earlier adolescence (31).

Presently, China has adopted the common international standard to determine precocious puberty, which is defined as presenting secondary sexual characteristics before age of 8 years (32). This definition originated from Europe and has been used for decades, and it includes 99% of the population with normal pubertal development and 1% of the population with abnormalities (33). However, based on this criterion, some single-center studies have shown that the prevalence of precocious puberty in Chinese girls is much higher than 1%. For example, a survey on the development of pubertal sexual characteristics in >10,000 primary school students from Shanghai in 2014 reported that the prevalence of precocious puberty in girls aged 6-7 years was 19% (34). Another survey in 2018 on the adolescent sexual development of >10,000 children aged 6-16 years in Shenzhen also reported that the proportion of girls with secondary sexual characteristics before age of 8 years was 11.86% (35). The high prevalence in above surveys collectively suggests that the standard definition of precocious puberty is inappropriate for the current situation of pubertal development of Chinese girls. The results of the Chinese Student Physical Fitness and Health Survey conducted every 5 years showed that the height of 18-year-old girls (near adult height) was still increasing steadily (36), indicating that although pubertal development advanced gradually, its duration did not shorten and adult height remained unaffected. This finding was further confirmed by a longitudinal study from Thailand, which indicated that the final average height of 104 girls age at thelarche of 7-9 years was similar to the average target height (154.0 ± 4.9 cm *vs*. 153.1 ± 4.8 cm) (37). The results of the above studies further revealed that it is normal for girls to develop early puberty. Although there is a lack of representative investigations on children’s pubertal development, the results of existing multicenter studies showed that B2 of Chinese girls were advanced at both median age and percentile ages. In a newly published multicenter large sample survey, the P_3_ age of B2 is 6.3 years, further confirming that pubertal onset age of Chinese girls has been significantly ahead of time. In 2020, a systematic review and meta-analysis proposed that the definition of sexual precocity should be re-examined for sexual development in advance worldwide and updated in at least some countries (5).

Global long-term trend studies on other secondary sexual characteristics of girls indicated that menarche and onset of puberty in girls were earlier than before (1). The average age of girls at menarche in China decreased by about 4.5 months per decade between 1985 and 2010 (38). Recall bias cannot be excluded because menarche age data were recorded *via* self-reports (5). The progression of secondary sexual characteristics in girls into puberty includes breast budding (thelarche) and development followed by pubic and axillary hair development, linear growth spurt, and menarche (1). There is still an early trend of girls’ puberty onset age in China based on breast development age. Thus, a reasonable definition is urgently needed to avoid misjudging normal children as precocious puberty, which might otherwise cause unnecessary social anxiety and excessive medical treatment. Altogether, normal age of breast onset is set at the age when 95% of girls present B2 (6, 39), which suggested that the puberty onset time of Chinese girls should be at P_3_ or P_5_ ages of B2.

## 5 Limitations

The present review has several limitations. First, there is a lack of data from surveys on adolescent development across the countries. Second, most studies did not provide detailed Tanner stage sample sizes or percentile values, and the current results showed that the B2 of Chinese girls was advanced, which made it impossible to calculate P_3_ or P_10_ cutoff age for B2. Herein, we aimed to provide data from China to support global efforts and determine the timing of pubertal development onset in girls. Nevertheless, we underscore the need to conduct further national or large-scale surveys to help accurately assess timing of the initiation of pubertal breast development among Chinese girls, and doing so will enable us to propose a new specific cut-off point more suitable for the current adolescent breast development among Chinese girls.

## 6 Conclusions

Taken together, our findings indicated that pubertal breast development age among Chinese girls presented an advanced trend over the past 20 years. As the precocious standards have significant implications for children, parents, and even the society, and the existing standard is already not suitable for the current girls in China. Therefore, it is necessary revisit and redefine “precocious puberty” and provides useful recommendations for clinical practice.

## Author contributions

WS and HL contributed to study design. WS, XZ and HL contributed to the systematic search, screening, and data abstraction. WS and HL contributed to writing the first draft. All the authors revised and approved the manuscript.

## Funding

This study was supported by The Special Fund of the Pediatric Medical Coordinated Development Center of Beijing Hospitals Authority (XTZD20180403), Public service development and reform pilot project of Beijing Medical Research Institute (BMR2019-11), and CAMS Innovation Fund for Medical Sciences (CIFMS) (2016-I2M-1-008).

## Conflict of interest

The authors declare that the research was conducted in the absence of any commercial or financial relationships that could be construed as a potential conflict of interest.

## Publisher’s note

All claims expressed in this article are solely those of the authors and do not necessarily represent those of their affiliated organizations, or those of the publisher, the editors and the reviewers. Any product that may be evaluated in this article, or claim that may be made by its manufacturer, is not guaranteed or endorsed by the publisher.
