# Supplementary material for: Secular trends in age at pubertal onset assessed by breast development among Chinese girls: A systematic review
Source: Front Endocrinol (Lausanne). 2022 Nov 24;13:1042122. doi: 10.3389/fendo.2022.1042122 (PMC9729541; doi:10.3389/fendo.2022.1042122)
Supplement: Supplementary file 1 [file Table_1.docx]

Supplementary Materials

**Supplementary Table S1: Literature search strategy**

| 1.Pubmed  (puberty OR pubertal OR adolescent OR adolescence) AND (sexual development OR breast development OR thelarche) AND (age) AND (girls) AND (China) 515  2. Cochrane  (puberty) AND (sexual development OR breast development) AND (girls) AND (China) 10  3.embase  'puberty' AND ('sexual development' OR 'breast development') AND 'girls' AND 'China' 89  4.cnki  SU%= pubertal AND (SU%= sexual development OR SU%= breast development) AND SU%=age 263  5.wanfang  SU:(pubertal) and (SU:(sexual development) or SU:(breast development)) and SU:(age) 1729  6.VIP  U= pubertal AND (U= sexual development OR U= breast development) AND U=age 495 |
| --- |
